# Ethnopharmacological Profile, Phytochemistry and Therapeutic Potential of *Aegle marmelos* L. for the Treatment of Neurological Disorders

**DOI:** 10.1155/jnme/2275526

**Published:** 2025-06-11

**Authors:** Ashwani Sharma, Dalapatghi Gugulothu, Tarun Virmani, Anjali Sharma, Girish Kumar, Kuldeep Singh, Divya Jain, Md. Shimul Bhuia, Raihan Chowdhury, Nowreen Tabassum Ahammed, Muhammad Torequl Islam

**Affiliations:** ^1^Delhi Institute of Pharmaceutical Sciences and Research (DIPSAR), Delhi Pharmaceutical Sciences and Research University, New Delhi, India; ^2^Amity Institute of Pharmacy, Amity University, Greater Noida, Uttar Pradesh, India; ^3^CDSCO, FDA Bhawan, New Delhi, India; ^4^Department of Pharmacology, Institute of Pharmaceutical Research, GLA University, Mathura, Uttar Pradesh, India; ^5^Department of Microbiology, School of Applied & Life Sciences, Uttaranchal University, Dehradun 248007, Uttarakhand, India; ^6^Phytochemistry and Biodiversity Research Laboratory, BioLuster Research Center Ltd., Gopalganj, Dhaka 8100, Bangladesh; ^7^Department of Pharmacy, Gopalganj Science and Technology University, Gopalganj, Dhaka 8100, Bangladesh; ^8^Department of Biology, Touro University, New York City, New York, USA; ^9^Pharmacy Discipline, Khulna University, Khulna 9208, Bangladesh

**Keywords:** *Aegle marmelos*, Alzheimer's disease, depression, epilepsy, Parkinson's disease

## Abstract

*Aegle marmelos* (L.) Corrêa, commonly known as the bael fruit tree, is a member of the Rutaceae family and holds significance in Ayurvedic herbal medicine due to its myriad therapeutic properties. This paper seeks to delve into the diverse benefits offered by the bael fruit tree, exploring various plant parts, including leaves, fruit, bark, and seeds, all of which contain bioactive compounds with therapeutic potential. The bael fruit, with its diverse phytochemical profile, exhibits potential health benefits ranging from radioprotection and antibacterial properties to antioxidant and hepatoprotective effects. Additionally, this review highlights the limited preclinical studies on AMs' efficacy in treating neurological disorders, emphasizing the need for more clinical trials to validate its potency and safety. Specifically, the effects and mechanisms of AM extract in addressing Alzheimer's disease, anxiety, depression, epilepsy and Parkinson's disease are explored. In conclusion, AM emerges as a plant of considerable nutritional and pharmacological value, with the potential to contribute significantly to the treatment of neurological disorders. Despite its promising attributes, the limited preclinical studies necessitate further clinical trials to confirm its efficacy. This review consolidates relevant studies, offering insights into AMs' ethnobotany, chemical constituents, pharmacological properties and potential application in neurological disorders. The comprehensive examination underscores the need for continued research to unlock the full therapeutic potential of this versatile plant.

## 1. Introduction

*Aegle marmelos* (L.) Corrêa, also known as Bilva in Sanskrit, is a widely used traditional medicinal plant in India. It is native to various regions such as Sri Lanka, India, Pakistan, the western Himalayas, Thailand, Malaysia and the Andaman Nicobar Islands. Numerous parts of the AM plant, including the leaves, roots, bark and fruits, are commonly used in the production of Ayurvedic pharmaceuticals and cultural medicines. These medicinal applications have been passed down through generations, and the plant continues to be an important part of traditional Indian medicine [1]. AM is known for its therapeutic properties, and many plant parts are utilized to treat a wide range of physical and mental ailments. Utilizing plants for therapeutic purposes has been practised for thousands of years, and it is documented in ancient Indian literature such as the Atharvaveda, Rigveda, Charak Samhita, Yajurveda and Sushrut Samhita. These ancient texts provide insights into the traditional use of plants in Indian medicine and highlight the importance of natural remedies for maintaining health and treating illnesses [2]. Researchers are conducting studies to identify the active chemical components of plants that have been traditionally used for medicinal purposes. By understanding the chemical properties of these plants, researchers hope to develop new treatments for various disorders.

Neurodegenerative diseases, including depression, epilepsy, Parkinson's disease and Alzheimer's disease, pose a serious threat to world health. Around 55 million individuals worldwide suffer from Alzheimer's disease alone, and by 2050, that figure is expected to rise to 139 million [3]. According to estimates, about 10 million individuals worldwide suffer with Parkinson's disease, and as the population ages, its incidence is predicted to quadruple by 2040. Over 280 million people worldwide suffer from depression, which is still a major source of disability, while about 50 million people worldwide have epilepsy, many of whom are still unresponsive to conventional therapies [4]. Despite these startling statistics, there are currently few effective treatment options available, and they are frequently accompanied by serious adverse effects. AM, a natural resource with remarkable neuroprotective potential based on its pharmacological characteristics and historic usage in medicine, is one example of the alternative treatment approaches that are desperately needed. By offering safer and more efficient treatment options, integrating such methods into contemporary therapies might alleviate the increasing burden of chronic illnesses [5, 6].

Over the last five decades, numerous studies have been conducted on these plants, using advanced scientific techniques. These studies have reported a wide range of medicinal properties associated with plant components including anticancer, antifungal, antidiabetic, antibacterial, hepatoprotective, antioxidant, haemolytic, anti-inflammatory, larvicidal and other activities.

### 1.1. Research Gap and Prior Studies

Although AM has long been used to treat a variety of illnesses, including neurological problems, thorough research confirming its effectiveness and clarifying its processes in treating particular neurological issues is lacking. Existing research on AM primarily focuses on preclinical models, where its extracts have demonstrated acetylcholinesterase (AChE) inhibitory activity, suggesting potential benefits for Alzheimer's disease. Aegeline, a compound isolated from AM, has shown protective effects against Parkinson's disease by mimicking the yeast soluble N-ethylmaleimide-sensitive factor attachment protein receptor (SNARE) protein Sec22p to suppress α-synuclein-induced toxicity [7].

Moreover, imperatorin-mediated peroxisome proliferator–activated receptor (PPAR)-γ activation has been linked to anticonvulsant efficacy in epileptic models. Notwithstanding these encouraging results, the majority of research is restricted to in vitro tests or animal models, and there are no significant clinical trials to validate the pharmacokinetics, safety, and effectiveness in people [8]. Furthermore, little is known about the particular molecular pathways that underlie AM neuroprotective properties, especially how it interacts with neurotransmitter systems like serotonin and GABA [9, 10]. There are also few comparisons with conventional therapies for neurological conditions like Parkinson's and Alzheimer's. Moreover, there has been limited research on developing advanced formulation and delivery mechanisms, such as brain-targeted nanoformulations, to enhance the bioavailability and targeted efficacy of AM [6].

This review aims to consolidate prior findings, highlight these gaps, and propose a framework for advancing research through systematic clinical evaluations and the development of innovative delivery systems, thereby bridging the gap between traditional knowledge and modern evidence-based applications.

The primary objective of this study is to evaluate the neuroprotective potential of AM by investigating its pharmacological properties, mechanisms of action, and therapeutic applications in the management of neurodegenerative illnesses. AM's phytochemical constituents that underpin its neuroprotective effects are examined in this review, along with how these properties might be applied therapeutically to address specific neurological disorders like epilepsy, Parkinson's disease, Alzheimer's disease, depression, and anxiety. It also points out current gaps in preclinical and clinical studies into AM's safety, effectiveness, and modes of action [6]. The paper also looks at how sophisticated drug delivery methods, such brain-targeted nanoformulations, might improve therapeutic efficacy for the focused treatment of neurological disorders. By discussing these topics, the review hopes to compile earlier findings, identify areas that need more research, and offer a framework for developing AM's therapeutic uses in neurology.

Through ongoing research, scientists are gaining a better understanding of the potential health benefits of plants and plant components, and this knowledge may lead to the development of new treatments for a variety of health conditions [11]. AM belongs to the family ‘Rutaceae' believed to be originated and is extensively available in India.  Table 1 displays the taxonomic categorization in many Asian nations. Some of the common names for this plant include Bael, Bengal quince, Golden apple and Wood apple. There may be other names used for this plant in different parts of the world as well. These different names reflect the widespread cultural and traditional uses of the plant in various regions. This is a significant medicinal plant with traditional and folk remedies, as well as ethnomedicinal uses. AM leaves are used to worship Lord Shiva and Goddess Laxmi so that AM is cultivated as temple garden plants. It is true that the fruits of the bael tree have been used as traditional medicine and food for a long time. Specifically, bael fruit has been used to treat diarrhoea and dysentery due to its potential antidiarrheal and antidysenteric properties. In addition, there is some traditional belief in certain parts of India that the leaves of the bael tree may cause abortion and infertility in women. However, this belief is not supported by scientific evidence [13]. Dysentery, malabsorption, dyspepsia, neurological illnesses, vomiting, oedema and rheumatism all are believed to be treated with bael leaves and fruits [1].

### 1.2. Geographical Distribution

The AM plant is a subtropical species capable of thriving at elevations up to 1200 m above sea level. It thrives in hilly as well as in plain places with dry woodlands. AM may grow in a variety of environments and can be cultivated all over the world. It is native to India, particularly the Eastern Ghats and Central India. Its use has been described in ancient manuscripts dating back to 800 B.C. In 1629 A.D., Hiuen Tsiang, a Chinese Buddhist pilgrim who visited India, also documented the presence of this tree [14]. It is planted all across India, and because of its mythological significance, it is primarily grown around temples. Bael tree is mainly found in central and southern India including the Deccan Plateau, the East coast, and sub-Himalayan regions from Jhelum to West Bengal. It can also be found in the Andaman Islands. The tree grows in several Indian states including Bihar, Jammu and Kashmir, Andhra Pradesh, Madhya Pradesh, Maharashtra, Punjab, Himachal Pradesh, Karnataka, Rajasthan, Kerala, Uttar Pradesh, Tamil Nadu and West Bengal [15].

**Figure 1:**
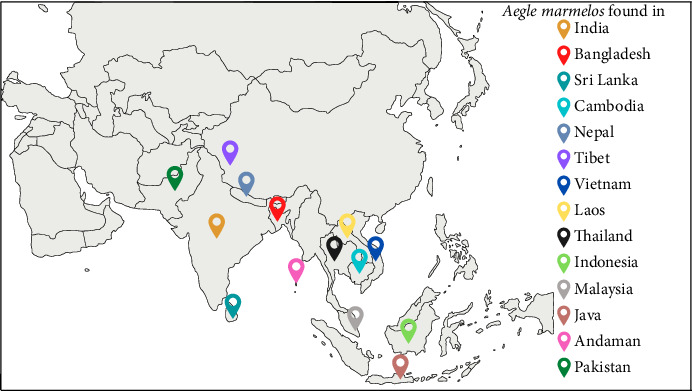
Geographical distribution of *Aegle marmelos* (L.) Corrêa in Asia.

According to the shape of the fruit, there are 13 different types of AM found in West Bengal. Fruits are divided into five groups: oval, spherical, oblong, flat and pear-shaped with different size subgroups (small, medium and large). Outside India, it is also grown in Myanmar, Nepal, Cambodia, Tibet, Vietnam, Laos, Sri Lanka, Ceylon, Bangladesh, Thailand, Indonesia, Malaysia and the drier parts of Fiji, Java, and to a lesser extent in the Northern Luzon of Philippine Island [1, 16, 17], the geographical distribution of AM is shown in Figure 1. In various regions, it is commonly known by the different regional names which are mentioned in Table 2.

### 1.3. Morphological Description

The bael tree is a deciduous tree with a compact or dense crown and no weeping branches. The lower limbs can droop at times. The tree is hardy and adapts well to a variety of soil and climatic conditions. It can reach a height of 10 m or more, with medium to massive limbs. The fruits are mostly found on the canopy's edge, and with narrow oval ends, the trunk is short and thick. The wood is stiff and slow growing, and a central pith can be found in young wood. The trees in natural settings are smaller and more uneven. It is a slowly growing, medium- to a small-sized tree that reaches a height of 25 to 30 feet. The stem is gentle and thick, with a few prickly twigs thrown in for good measure [19, 20]. The comprehensive morphological features of AM are detailed in Table 3.

## 2. Ethnobotanical Uses of AM

Various components of AM are employed by numerous Indian Ayurveda practitioners and traditional herbal healers for addressing a range of health issues. Every part of the plant is utilized to make a variety of medicines. Fruit is one of the most significant for curing a wide range of disorders. Various varieties of powder, paste and tablets derived from the bael are utilized. Bael is a significant element in the creation of dasamula, chyavanprash and other dishes. It is used to treat a variety of ailments due to its carminative and digestive effects. In Ayurveda, bael is used to cure chronic diarrhoea and dysentery and as a brain tonic, among other things [23, 24].

Twice daily, AM leaf extract (AME) is taken to treat intestinal worms, ulcers and ophthalmia. Eye diseases are treated with a poultice made from the bael leaves. The leaf extract has several medicinal uses, one of which being the treatment of diabetes [18]. Additionally, those who have a fever or cold are administered a decoction of bael root. Fevers and heart problems can be treated with root and bark decoction [25]. Ethnobotanical uses in different conditions along with the parts used are shown in Table 4.

### 2.1. Nutritional Composition and Phytoconstituents (Bioactive Compounds) of AM

In terms of nutrition, bael fruits are high in water (60%–65%), sugar (11%–17%), fibres (5%) and a good amount of carbohydrates (9%–21%). 1.7% of the bael fruit pulp is made up of minerals including copper, iron, calcium, potassium and phosphorus. The fruit is also loaded with nutrients, including riboflavin, thiamine, ascorbic acid and vitamin A [29]. AM has a higher calorific value than mango, apple and guava. In addition to sucrose, fructose and glucose, AM also contains galactose, arabinose, rhamnose, xylose, threose and galacturonic acid. Linoleic, linolenic, myristic, oleic, palmitic, ricinoleic and stearic fatty acids make up the fats found in AM [30]. The various components of AM are home to a diverse range of bioactive chemicals. Marmelosin is very abundant in the unripe fruit, with reported weight concentrations ranging from 0.4% to 0.6% [31].

The fruit and leaves have been found to contain alkaloids such aegeline, skimmianine and marmeline; the concentrations of aegeline in the leaves range from 0.15 to 0.25 mg/g, while those in the fruit range from 0.10 to 0.18 mg/g [32]. The amounts of coumarins, such as psoralen and imperatorin, in the root and bark range from 0.01% to 0.05%. For example, the amount of marmelosin in bael fruit varies from 415.75 to 737 μg/g, and the total phenolic content (TPC) varies from 10.6 mg GAE/g to 25.14 mg GAE/g, depending on drying conditions. There have also been reports of significant levels of carotenoids like β-carotene (51.67–153.43 μg/100 g) and coumarins such as imperatorin and marmelosin [33]. Aegeline (0.15–0.25 mg/g) and skimmianine are two alkaloids that enhance its pharmacological activity. The pharmacological qualities of the essential oils extracted from the leaves are further enhanced by the presence of 10%–15% limonene and 20%–25% linalool [33].

The therapeutic significance of AM is highlighted by these quantitative insights into the phytochemical composition, which also serve as a basis for additional pharmacological research [17]. The bulk of studies on the isolation and characterization of chemicals has been recorded by several Indian researchers. Extensive research has been done on various portions of AM, and as a result, many groups of compounds such as alkaloids, amino acids, coumarins, fatty acids and terpenoids have been identified from its various components [34]. Flavonoids, phenolics, carotenoids, alkaloids, polysaccharides and terpenoids are plant secondary metabolites that contribute to flavour, colour and health-promoting properties [35]. The abundance of phytochemicals in AM accounts for its multiple health advantages. A variety of bioactive chemicals were found in the leaves including alkaloids, flavonoids, phenols, saponins and tannins [36, 37]. Isolated chemical compounds found in a particular part of the plant with the specific medicinal property of AM are shown in Table 5.

The main bioactive substances and their pharmacological actions in AM fruit are collected in Table 6. Numerous therapeutic benefits, including neuroprotection, antioxidant activity, antidiarrheal, antidiabetic, hepatoprotective, radioprotective and anticancer activities, are facilitated by these substances [17, 58]. Antioxidant qualities, enzyme inhibition, calcium channel blockage and glucose metabolism modification are some of the possible processes exhibited by the bioactive chemicals found in AM [33]. This wide range of actions demonstrates the plant's substantial medicinal promise, especially for the treatment of long-term illnesses including diabetes, liver disease, cancer and neurological problems. To confirm these results and create practical applications, more research is required [17].

## 3. The Rationale Behind the Selection of AM for Neurological Disorders

AM is a medicinal plant commonly used in traditional medicine systems, particularly in Ayurveda, for various health conditions including neurological disorders. The rationale behind choosing AM as an herbal medication against other herbal drugs and allopathic medications for neurological diseases. It is generally considered safe and well-tolerated when used in recommended doses. It has a low risk of adverse effects compared to some other herbal drugs and allopathic medications used for neurological disorders [42]. It has been shown to possess neuroprotective, anti-inflammatory, antioxidant and anticonvulsant properties, which may be beneficial for the treatment of neurological diseases. Several studies have reported its effectiveness in improving cognitive function, reducing oxidative stress and attenuating neurodegenerative processes [59].

It is widely available and can be easily cultivated in many regions, making it a cost-effective and accessible option for those seeking alternative treatments for neurological disorders. It has a long history of traditional use in Ayurveda and other traditional medicine systems for the treatment of various ailments including neurological disorders. Its use in traditional medicine suggests that it may have therapeutic properties that have been validated by generations of experience [17]. It is worth noting that while AM may have potential benefits for neurological disorders, it is important to consult a healthcare professional before using it as a treatment.

## 4. Methodology of the Review

In this review, the specific neurological disorders have been focused on and the aspects of AM have been explored for its ethnobotany, easy availability and its potential for numerous ailments. A literature search was conducted: Used PubMed databases to search for relevant studies on AM and its potential use for neurological disorders. Used keywords such as ‘*Aegle marmelos*', ‘neurological disorders', ‘ethnopharmacology' and ‘traditional medicine' for which nearly 122 articles found in the database which have been published in the recent years. Most relevant studies were evaluated by reading the abstracts and full-text articles of the studies which help to find and determine that they are relevant and useful for the review. The studies that provide information on the ethnobotany, chemical constituents of AM, its pharmacological properties and efficacy in treating neurological disorders have been chosen. Once we had gathered enough information, the review was organized it into a coherent structure. This may involve creating an outline and grouping similar information and started with an introduction about the AM that provides background information and context for the review, followed by a section on the ethnopharmacological profile of AM, its chemical constituents, structures and its pharmacological properties. Next, describe the potential use of AM for neurological disorders including its mechanism of action. Finally, concluded the review with a summary of the main findings and recommendations for future research.

## 5. Neuroprotective Activities of AM

Due mainly to their anti-inflammatory and antioxidant properties, a number of AM's bioactive components have demonstrated notable neuroprotective benefits. For example, flavonoids with antioxidant qualities, like quercetin and rutin, have been shown to shield neurons from oxidative stress, which is a primary cause of neurodegenerative diseases like Parkinson's and Alzheimer's. By scavenging free radicals, these flavonoids lessen inflammation and neuronal damage in the brain [25, 60].

Similar to this, it has been demonstrated that the coumarin molecule marmelosin inhibits AChE, a crucial mechanism for enhancing cognitive function and managing Alzheimer's disease. Marmelosin improves neurotransmission and lessens Alzheimer's disease symptoms by raising acetylcholine (ACh) levels through AChE inhibition [61, 62]. The fruit contains an alkaloid substance called aegeline, which has demonstrated promise in regulating neurodegenerative processes, especially in Parkinson's disease. It has been shown to replicate the functions of the yeast SNARE protein, which helps stop the aggregation of proteins that are typical of Parkinson's disease pathology, and reduce α-synuclein-induced toxicity [7].

Furthermore, a terpenoid called linalool has shown anti-inflammatory and neuroprotective properties, which may enhance cognitive performance and provide therapeutic advantages in the treatment of anxiety-related conditions. Its involvement in neurological health is supported by its capacity to control oxidative stress and decrease inflammation [63]. In addition to showing encouraging outcomes in preclinical research, these particular phytochemicals from AM may have uses in the treatment of neurological conditions. To confirm their effectiveness and investigate their clinical value, more investigation is required, including clinical studies.

### 5.1. AME: Implications for Alzheimer's Disease Treatment

AD is a progressive neurological disorder known for a gradual memory loss, cognition and behavioural problems. It is a leading cause of death in elderly patients. Significant cholinergic neuron loss, geriatric plaque composed of Abeta protein and neurofibrillary tangles of actin filaments protein tau are pathological markers of AD [64–67]. There are still few effective therapies available despite tremendous advancements in our understanding of the biology and aetiology of AD. A characteristic symptom of AD patients is significant cholinergic neuron loss, notably within the frontal cortex which is followed by a shortage of ACh, a neurotransmitter found in the cerebral cortex junctions [67–69]. The death of cholinergic neurons as well as the resulting drop in ACh levels has been linked to cognitive impairment in Alzheimer's patients [70, 71].

Consequently, elevating ACh levels through the inhibition of the enzyme AChE, responsible for breaking down ACh, seems to alleviate the symptoms of cognitive loss in AD, and this approach holds promise for the development of medications. Only three inhibitors for cholinesterase have been licensed by the US FDA to treat AD such as donepezil, galantamine and rivastigmine, and drugs have some severe side effects [72, 73]. These medications have helped patients with AD manage their symptoms and function better, but none of them can stop or reverse the disease's course altogether [74].

A large body of evidence suggests that oxidative stress has a role in the aetiology and pathogenesis of AD. It has been shown that oxidative stress, which results from an imbalance between the production of free radicals and the antioxidant system's ability to eliminate them, causes reactive oxygen species (ROS) and other free radicals to accumulate and cause cellular and molecular abnormalities in sporadic AD [75–77]. Although the specific processes causing these harmful consequences are unknown, oxidative stress is known to occur before the production of neurofibrillary tangles and senile plaques, two hallmarks of AD [78, 79]. It was also discovered that Abeta protein, which is a significant component of senile plaque in AD patient's brains, induces an increase in the production of free radicals in neuronal cells, resulting in oxidative stress and death of cells [80, 81].

Asaduzzaman et al. 2014 worked on AM, which is popular in Ayurveda, or traditional Indian medicine describes the medicinal characteristics of AM in great detail [61]. AM leaf has a legendary reputation for boosting intelligence and memory [82]. As per the hypothesis proposed by Asaduzzaman et al. in 2014, the noteworthy abnormality observed in AD is the potential of AME to counteract the reduction of ACh in the hippocampus (HIP) and cortex of the brain. As a result, increasing ACh levels inside the cleft of synapses by inhibiting AChE, which is implicated in ACh breakdown, is a well-accepted therapy method for AD. ROS, which is produced during oxidative stress, is thought to cause cellular and molecular abnormalities in sporadic AD, according to a large body of research [75, 76, 83]. Hydroxyl radicals are the most common ROS and are extremely hazardous; their contribution is significant to oxidative stress and are recognized to be detrimental to neurons in AD [84].

The findings demonstrated that all of the extracts had the potential to scavenge hydroxyl radicals. Lipid peroxidation is the process by which ROS attack lipids, forming a carbon radical that interacts with oxygen to generate a peroxyl radical, culminating in lipid peroxides [85, 86]. The brain is a major target of oxidative stress because of its high lipid content and extremely high concentration of polyunsaturated fatty acids, which are particularly vulnerable to oxidation. Increased levels of lipid peroxidation products, such as 4-hydroxynonenal or 2-propenal, have been seen in the brains of people with AD, as well as increased lipid peroxidation in the cerebrospinal fluid and plasma [87]. Thiobarbituric acid can be used to assess lipid peroxidation products [88, 89].

**Figure 2:**
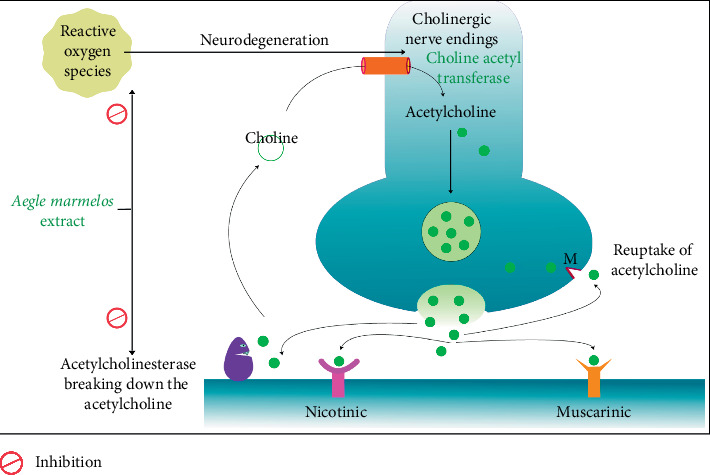
Diagrammatic representation of *Aegle marmelos* (L.) Corrêa inhibiting ROS and cholinesterase resulting in an availability of more acetylcholine which attenuates AD.

It has been documented that the extract of AM leaf showed anti-AChE activity concerning its concentration and also showed that antioxidant activity estimated by reducing power assay, scavenging DPPH radicals, quenching hydroxyl radicals and inhibiting lipid peroxidation is a key activity associated with the extract. It is evidenced that AM suppresses AChE activity and a few aspects of the oxidative stress network, all of which can lead to Alzheimer's pathogenesis. As a result, the extract could believe to have the potential to be a safe treatment for AD [59, 61]. The mechanism of AM is shown schematically in Figure 2.

### 5.2. AME: Implications for Parkinson's Disease Treatment

PD is a neurological movement disorder. The first sign may be a barely detectable tremor in only one hand [6]. Tremors are common, but they are usually accompanied by stiffness or decreased movement. Early on in the course of PD, the face may have little or no expression. The arms might not swing when a person walks [90]. The speech of the person may become muffled or slurred as a result of this. As the condition progresses, the symptoms of PD worsen [91].

Protein transport from the endoplasmic reticulum (ER) to the Golgi body depends on the anterograde and retrograde vesicle membrane fusion processes facilitated by the SNARE or ySec22p [61, 92–94]. Specific fusion processes between vesicles are mediated by different SNAREs. The ultimate destination of a protein is dictated by the SNARE that is involved. The helix bundles are in charge of causing membrane fusion. These are made up of vesicle-localized v-SNAREs and targeting-localized t-SNAREs. According to a new study, yeast transport from the ER to the Golgi complex is greatly aided by the interaction between the proteins Bet1 and Bos1 and the v-SNARE ySec22p [95]. SNARE proteins are members of a protein family that are substantially conserved across yeast to human beings. Humans contain three Sec22 proteins: hSec22Bp, hSec22Ap and hSec22Cp, but yeast only has one, ySec22p [96]. 20% similarity with hSec22Ap (NCBI Accession #XP 011510978.1), 37% similarity with hSec22Bp (NCBI Accession #NP 004883.3) and only 20% homology with hSec22Cp (NCBI Accession #AAQ89400.1) are shown by the ySec22p (NCBI Accession #KZV09512.1). A surplus of human α-synuclein (α-syn) is thought to be responsible for PD-related neuronal death [97, 98].

**Figure 3:**
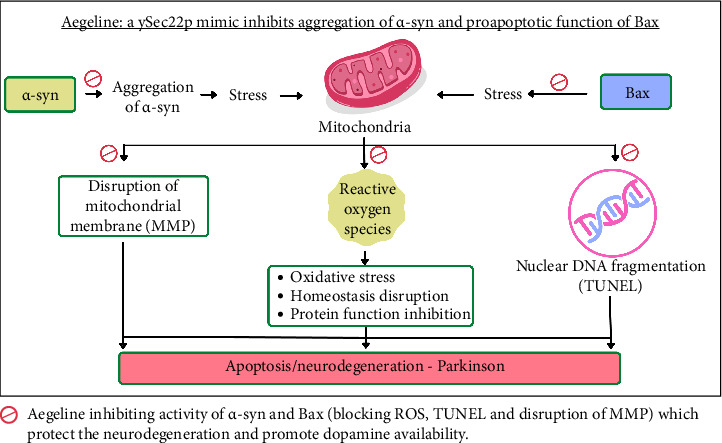
Aegeline mimicking the ySec22p holds the potential to impede yeast apoptosis triggered by α-syn aggregation and Bax expression. The expression of human proteins α-syn and Bax in yeast illustrates potential mechanisms that could induce apoptosis. The abbreviations MMP, ROS and TUNEL represent mitochondrial membrane potential, reactive oxygen species and terminal deoxynucleotidyl transferase dUTP nick end labelling, respectively.

In yeast, an excess of α-syn leads to cell death through apoptosis [99, 100]. Increasing the expression of ySec22p and two other SNAREs, Ykt6p and Bet1p, which are essential parts of the ER-to-Golgi transport pathway, was shown to be an effective way to reverse the growth inhibition caused by α-syn expression in yeast cells. On the other hand, α-syn appears to cause toxicity in mammalian cells by opposing ER/Golgi SNAREs, which causes a delay in the transit of proteins from the ER to the Golgi [101]. A new idea implies that hSec22p/hSec22A/hSec22Bp, the human homologs of ySec22p, might be expressed under transcriptional regulation as a means of preventing synucleinopathies through the inhibition of α-synuclein-mediated toxicity [7]. All the above scenarios lead to cause Parkinson's by gradual loss of dopaminergic neurons, leading to reduce the level of dopamine as shown in Figure 3.

Derf et al. 2019, the study highlights aegeline, identified as a chemical constituent isolated from AM leaf, and its impact on a mechanism observed. It was found that aegeline that inhibits the activity of SNARE protein ySec22p and eventually inhibits the apoptosis in yeast-induced PD by both α-syn and Bax. These two human proteins play crucial roles in neuronal apoptosis in PD. Molecular modelling supports the validity of the experimental findings. The yeast-based assay indicates that aegeline holds evidence for potential therapies in PD [7].

### 5.3. AM Implications for the Treatment of Epilepsy

Epilepsy believed as a chronic illness that has high mortality and morbidity rate. It has a global impact on a large number of people [102]. The key pathogenetic mechanisms driving epilepsy are known to be an increase in cholinergic and glutamatergic pathway activity and a decrease in GABAergic neurotransmitters at the molecular level [103, 104]. Antiepileptic medications (AEDs) include succinimides, aliphatic carboxylic acid, hydantoins, iminostilbenes and newer antiepileptic pharmaceuticals developed in recent decades such as gabapentin, felbamate, vigabatrin, lamotrigine and others are used in the clinical care of epilepsy [105, 106]. Moreover, current epileptic drug therapy is hampered by a slew of side effects, including cognitive and behavioural symptoms, hypersensitive responses, teratogenic possibility and toxicity over time, and side effects are a considerable issue because treatment typically lasts several years [107]. Furthermore, AEDs increase depression associated with epilepsy, which leads to low self-regard and an inferior lifestyle, which impacts seizure control clinical outcomes [8]. As a result, additional therapeutic strategies for treating epilepsy must be explored and developed.

Some relevant literature shows that PPARs are a group of nuclear receptors that control a range of physiological processes. PPAR-c is important in neuroinflammation and lipid metabolism [94, 108–110]. The HIP [111], the basal ganglia [112] and microglia, and astrocytes [112] have all been implicated as sites where PPAR-c may be expressed. In the brain, PPAR-c activation has been shown to suppress inflammatory responses [113–116]. PPAR-c activations have also been shown to reduce proinflammatory mediators, preventing the production of inducible nitric oxide synthase (iNOS) in brain cells [117–119].

Nitric oxide (NO) is indeed a neurotransmitter implicated in several physiological mechanisms that exist within the brain, including extended potentiation and memories and pain sensory abilities [120–122]. In pathological situations such as epilepsy, AD, cerebral ischemia and PD, NO plays a role [113]. In pentylenetetrazole-induced (PTZ-induced) seizures, NO expression is elevated [123]. NO is also involved in the development of oxidative stress in the brain because it produces highly reactive peroxynitrite free radicals [124]. Production of nitric acid inhibition has been postulated as a potential method of PPAR-c ligand protection in many studies [109]. The protective effect of AME was shown to be reversed after pretreatment with L-arginine, a precursor of NO. As a result, the defence provided by AME therapy against PTZ-induced convulsions may be mediated by suppression of the NO pathway, which could be attributable to PPAR-c activation.

**Figure 4:**
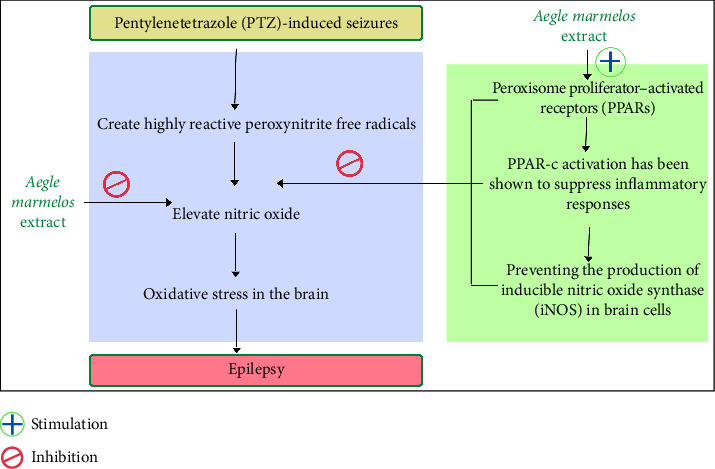
Showing the antiepileptic activity of AME by both PPAR-c activation and nitric oxide pathway inhibition.

According to the study done by Singh and Goel, the leaves of AM were tested for anticonvulsant activity in mice suffering from PTZ and maximum electroshock (MES)–induced convulsions. The main models for acute seizures are PTZ-generated convulsions and MES-induced convulsions. To induce depression in mice, repeated submaximal doses of PTZ were given that were adequate to cause convulsions with low mortality [125]. In this work, Singh and Goel employed similar models to assess the AME's ability to prevent both acute and persistent post-traumatic depression. The study findings show that treating rats with AME delays the onset and shortens the duration of tonic–clonic convulsions in a dose-dependent manner in a PTZ-induced model. Furthermore, AME medication reduces the duration of symptoms in MES-induced seizures and the impact is comparable to that of the conventional medicine phenytoin at greater doses. The protective effect of AME was shown to be reversed when the mice were pretreated with a PPAR-c inhibitor, indicating that it may be a PPAR-c-mediated action. Furthermore, in order to investigate the potential role of the nitric oxide pathway in this phenomena, the observed impact was reversed when L-arginine, a precursor of NO, was given before to AME. Remarkably, L-arginine and N-nitro L-arginine methyl ester hydrochloride (L-NAME), an inhibitor of NOs, had no effect on the ameliorative effects of AME. These data support the idea that AME's action is mediated by both PPAR-c activation and nitric oxide pathway inhibition as shown in Figure 4.

Imperatorin (coumarins derivative) isolated chemical constituent from AM has been reported for its antiepileptic activity in animal models and shows that AME therapy can help reduce the intensity of both MES- and PTZ-induced seizures. This protective effect could be due to the activation of PPAR-c activation, which suppresses NO generation further. In mice, chronic AME therapy is beneficial in reducing postictal depression [126].

### 5.4. AME Implications for the Anxiety and Depression Treatment

Anxiety and depression are the two most frequent stress-related mental illnesses, both of which can lead to disability and death (premature) [127]. Despite flaws, the monoamine concept of anxiety and depressive symptoms is widely accepted [128]. In addition to depression, most antidepressant medicines have been demonstrated to be beneficial against anxiety disorders [129]. Advances in neuroscience reveal that, in addition to monoamine deficiency, disruption of the GABAergic system plays a role in the pathophysiology of anxiety and depression [130]. Antidepressant-like GABA-B receptor antagonists additionally have been demonstrated to boost 5-hydroxytryptamine and dopamine neurotransmission [131, 132].

Synthetic medications used to treat anxiety and depression have a variety of side effects, including drowsiness, ataxia, sleeplessness and libido with benzodiazepines and selective serotonin reuptake inhibitors [133]. Drugs derived from natural sources are thought to have fewer negative effects while performing similarly to their synthetic equivalents in terms of curing illnesses. The hunt for innovative psychiatric pharmacotherapy from medicinal plants has recently developed dramatically, revealing the pharmacological efficiency of several plant species in several animal models [134].

The EPM is a well-established paradigm for examining anxiety-like responses in rodents that has a longstanding history. The model is based on rats' inherent aversion to open spaces (for fear of falling off). Rodents do not like to be in places that are open and bright because they can be seen by predators. Instead, they prefer to be in darker, enclosed spaces where they can hide and feel safe. Due to inconsistencies in anxiolytic compound results and a desire for more aimed therapeutic approaches, scoring additional, ethologically relevant behavioural indicators (such as head dips and stretch attend postures) may provide more sensitive measures of the effects of new anxiolytic compounds. This ethological method has improved the value of the plus-maze as a tool for studying anxiolytic action by removing locomotor confounds [135, 136].

Kothari et al. 2010 worked on AME to evaluate its antianxiety and antidepressant activity and reported that the EPM test revealed that AME reduced anxiety levels. Administering doses of 150 and 300 mg/kg of AM resulted in increased activity on the open arms of a maze, as the subjects spent more time there and made more entrances onto the open arms. Additionally, the rats displayed less risk assessment behaviour, as evidenced by a decrease in the number of stretch attend postures and head dips. Anxiolytic properties (300 mg/kg) of AME were comparable to those of common anxiolytics such as imipramine, diazepam and fluoxetine [137].

A popular animal model for assessing the effectiveness of antidepressants is the TST. It works on the principle that after a short fight, mice that are hanged upside down exhibit unusual immobility behaviour. This behaviour is suggestive of a depressive state that can be lessened by a number of medications that are well-known for their ability to treat depression in people [138]. In this study, mice were given methanol extract of AM leaves at doses of 150 and 300 mg/kg 1 h before being subjected to tail suspension test and the anti-immobility action was dosage-dependent. At 300 mg/kg, the antidepressant effect of AM was comparable to imipramine and fluoxetine [137].

The combination of a dose of AM (75 mg/kg) with a dose of diazepam (5 mg/kg) had no significant anxiolytic activity, implying that the methanol extract of AM leaves did not amplify the diazepam effect in EPM test. The anxiolytic activity of diazepam has been widely studied and confirmed to be related to its GABA facilitatory impact on GABAA receptors [139, 140]; however, the results of this investigation reveal that GABAA receptors are not involved in the anxiolytic activity of AM. Similarly, AM (75 mg/kg) combined with either fluoxetine (5 mg/kg) or imipramine (5 mg/kg) resulted in a dose-dependent increase in open-arm activity, a decrease in risk assessment behaviour in the EPM, and a shorter period of immobility in mice conducting the TST after a single dosage. These findings imply that AM improves imipramine and fluoxetine's anxiolytic and antidepressant effects [141].

Imipramine inhibits the reuptake of noradrenaline and serotonin, increasing their availability in the synaptic and thereby increasing adrenergic and serotonergic neurotransmission [142]. Fluoxetine is a serotonin reuptake inhibitor that helps serotonergic neurotransmission. The favourable effect of catecholamine and 5-hydroxytryptamine in EPM and TST appears to be attributable to the enhanced availability of these neurotransmitters at the postsynaptic receptor sites, as these neurotransmitters are implicated in the genesis of anxiety and depression. The fact that AM has anxiolytic and antidepressant effects at a subeffective level when combined with imipramine or fluoxetine suggests that it is involved in boosting monoamine levels at postsynaptic locations [137].

In the recent study, by reducing depressive-like behaviours in the forced swim test (FST), TST, open field test (OFT) and sucrose preference test, the hydroalcoholic extract of AM leaves significantly demonstrated an antidepressant-like effect in chronic unpredictable mild stress (CUMS) challenged rats. In the rats' HIP and prefrontal cortex (PFC), AME mitigated the changes in serotonergic systems, mitochondrial function and the generation of proinflammatory cytokines and dramatically decreased the CUMS-induced hyperactivity of the hypothalamic–pituitary–adrenal (HPA) axis. EAM can be extrapolated as a potential antidepressant-like effect of this approach in the preclinical study based on the findings [9]. Hence, it is evidenced that AME had substantial anxiolytic and depressive properties, presumably due to increased monoamine levels at postsynaptic regions. Therefore, it could be used as a natural psychotherapeutic agent in the treatment of stress-related disorders like depression and anxiety. However, a deep research and clinical trials are required for any conclusion.

A summarized presentation of chemical compounds responsible for neuroprotective activity with chemical structure and mechanism of action is given in Table 7.

## 6. Discussion of Findings

According to previous studies, AM is a medicinal herb that has shown promise in treating various illnesses. In particular, extracts from different parts of the plant and certain isolated compounds have demonstrated potential in treating neurological impairments. Research has found that AM may be beneficial for individuals with AD due to its ability to inhibit the activity of AChE and its antioxidant properties. A compound called aegeline, found in AM, has been shown to protect against PD by preventing cell death and reducing stress. Aegeline has also been found to alleviate depression by reducing stress levels and inhibiting the activity of MAO-A. Another compound in AM, called imperatorin, has been found to reduce the symptoms of epilepsy by activating PPAR-c and suppressing NO generation. Imperatorin has also been shown to alleviate depression by increasing 5-HT levels and preventing depressive-like behaviour in adolescents. Although most studies have been conducted on animals, these findings suggest that AM may be a promising agent for treating neurological diseases in preclinical research. However, more detailed research and clinical trials are needed to draw strong conclusions. In findings, this review suggests that AME could be used as a potential neuroprotective agent.

## 7. Conclusions and Future Perspective

Nowadays, herbal experts are focusing on finding natural remedies to avoid the side effects of synthetic drugs. One important medicinal herb that has been widely used in traditional medicinal systems like Ayurveda is AM. This plant can be found in different parts of India, and various forms such as fruit, leaf, bark, seed and root are used to cure different ailments.

The extract of AM has been reported to treat diseases such as ulcers, intestinal worms, eye disorders, diabetes, chronic diarrhoea and dysentery and also acts as a brain tonic. However, the management and treatment of neurodegenerative diseases require long-term therapy that can lead to severe side effects of drugs. Therefore, AM is suggested as a potential option for the management of neurodegenerative diseases without severe adverse effects.

This review specifically focuses on the effect of AM as a possible therapeutic option for anxiety, AD, depression, epilepsy and PD. The findings suggest that AM may have neuroprotective potential in treating various neurodegenerative diseases safely and effectively in preclinical research. AM may improve health with its other nutritional values after deep analysis by human clinical trials. However, more research is needed to explore the potential of AM in treating these conditions and to understand the underlying mechanisms of action. Overall, the literature suggests that AM could be a safe and effective approach for the management of neurodegenerative diseases derived from natural resources. It may be inferred that AM has the potential to attenuate neurological diseases through a variety of pathways based on the discussion and findings in this study. It will be a great opportunity for the researchers to take everything into account and move forward with the in-depth investigation and clinical trials. In the above study, researchers use extracts from various plant parts and deliver the dose conventionally. Future studies will have a fantastic opportunity to develop brain-targeted nanoformulations containing the therapeutic constituent for a specific ailment or an extract of AM. In recent trends and advancements, researchers are working on nanoformulations carrying both conventional and herbal drugs for the codelivery approach, which is quite effective and successful in terms of target specificity, bioavailability, efficacy and safety. For future prospects, the codelivery of AM with conventional drugs in brain-targeted nanoformulations would be a great approach [145].

## Figures and Tables

**Table 1:** Taxonomical classification of AM.

Kingdom	Plantae	Reference
Subkingdom	Tracheobionta	[12]
Superdivision	Spermatophyta	
Division	Magnoliophyta	
Class	Magnoliopsida	
Subclass	Rosidae	
Order	Sapindales	
Family	Rutaceae	
Genus	Aegle	
Species	*Aegle marmelos*	

**Table 2:** Common parlance names of *A. marmelos* in different languages.

Language	Common parlance names	Reference
Old Hindi	Sir Phal	[18]
Sanskrit	Shreephal, bilwa, bilva	
Malay	Pokok maja batu	
Tamil	Vilva pazham, vilva maram	
Telugu	Maredu	
Javanese	Modjo	
English	Indian bael, golden apple, holy fruit, elephant apple, bael fruit, Bengal stone apple, quince, Indian quince	
Indonesian	Mojo tree	
French	Oranger du malabar	
Khmer	Banu	
Latin	*A. marmelos*	
Bengali	Shreefal, bel	
Thai	Matum, mapin, tum	
Lao (Sino-Tibetan)	Toum	
Burmese	Opesheet, ohshit	
Marathi	Kaveeth	
Urdu	Bel	
Nepali	Gudu, bel	
Vietnamese	Trai mam, mbau nau	

**Table 3:** Morphological description of *Aegle marmelos* (L.) Corrêa.

Parts of plant	Morphological description	Reference
Leaves	Highly specific trifoliate leaves with a pointed tip and a circular base. The immature leaves are light green, but as they age, they turn a dark green colour.	[21, 22]
Fruits	The fruit has a rigid outer covering and is 5 to 12 cm in diameter. When unripe, it is green but when fully mature, it develops a yellowish-brown colour.	
Bark	The bark is dark or grey and has several long, straight spines.	
Flowers	The flowers are bisexual and greenish or yellowish in appearance. Fresh leaves are usually the first to show signs of flowering.	
Seeds	The seeds are petite, measuring approximately 1 cm in length, with a sturdy, flattened-oblong shape and a fuzzy coating. Additionally, they are encased in an adhesive sac.	

**Table 4:** Ethnobotanical uses in different conditions along with part used.

Part used	Ethnobotanical uses in different conditions	Reference
Whole plant	Alleviation for backache, treatment for dog bites, relief for breast pain, management of cholera, assistance with constipation, mitigation of convulsions, easing of cramps, diabetes control, remedy for diarrhoea, treatment for dysentery, relief for eye complaints, solution for gastric trouble, aid for abdominal disorders, management of jaundice, fever alleviation, use as a laxative, mitigation of nausea, treatment for snakebite, resolution of stomach disorders, support for heart disorders, alleviation of vomiting, tonic properties, assistance with night fever and healing for cuts and wounds.	[17, 20, 24, 26–28]
Fruit	Constipation relief, diarrhoea and dysentery treatment, epilepsy management, laxative and tonic use, assistance with digestive and gastric problems, stomachic properties, heart and brain tonic effects, ulcer treatment, intestinal parasite treatment, gonorrhoea management and antiviral qualities can all be found in this remedy.	
Leaf	Treatment for lesions, alleviation of backache, remedy for eye ailments, addressing abdominal abnormalities, relief from nausea, healing for scratches and wounds, management of ulcers, assistance for dropsy, correction of folic acid deficiency, support for heart weakness, remedy for cholera, control of diarrhoea, cardiotonic properties, regulation of glucose levels, treatment for animal traumas, management of neurological disorders, use as a hair tonic, relief for respiratory diseases, aid during childbirth, wound care, control of worms, provision of fodder for sheep, goats and cattle, and stimulation of breathing and contraction of the denervated nictitating membrane in anaesthetized cats—all are instances of applications in veterinary medicine.	
Root	Gastrointestinal problems, antiamoebic properties, treatment for dog bites, hypoglycaemic effects, assistance with heart problems, management of irregular fevers and alleviation of symptoms associated with rheumatism.	
Flower	Management of epilepsy and functioning as an expectorant.	
Root, bark	Used to treat fish poison	
Seed	Antipyretic	

**Table 5:** Isolated chemical compounds found in a particular part of the plant with the specific medicinal property of AM.

Chemical compounds	Isolated chemicals	Part of plant	Medicinal property	Reference
Alkaloids	Anhydromarmeline, aegelinosides A, aegelinosides B, ethyl cinnamate, halfordinol, marmeline, N-2-methoxy-2-[4-(3′,3′-dimethylallyloxy) phenyl] ethyl cinnamate, N-2-ethoxy-2-ethylcinnamide and O-3,3-(dimethylallyl) halfordinol	Fruits and leaves	Antidiabetic, antibacterial, anti-inflammatory and anticancerous	[38–43]
Terpenoids	Caryophyllene, cineol, cis-limonene oxide, cis-linalool oxide, cubedol, elemol, epicubebal, hexanylhexanoate, humulene, isosylvestrene, limonene, linalool, methyl perilate, myrcene, P-cymene, terpinolene and valencene	Fruit, leaf and bark	Anticancer, antiulcer	[44, 45]
Vitamins	Ascorbic acid, thiamin, niacin and riboflavin	Mainly fruit	Nutritional	[39]
Coumarins	Alloimperatorin, marmelosin, marmesin, methyl ether, scopoletin, scoparone, psoralen, marmelide, imperatorin, marmin, umbelliferone and xanthotoxol	All parts	Antidiabetic, antioxidant, anti-inflammatory, antidepressive, anxiolytic, antibacterial, antihelminthic and antianalgesic	[18, 20, 24]
Tannins	4,7,8-trimethoxyfuro-quinoline	Unripe fruit	Astringent, antidiarrhoea	[30, 46]
Carbohydrates	Arabinose, aralrinose, D-galacturonic acid, galactose, uronic acid and L-rhamnose	Bael fruit flesh	Source of energy	[47]
Flavonoids	Flavone-3-ols, flavone, flavone glycosides and rutin	Fruit	Hepatoprotective	[48, 49]
Fatty acids	Linoleic acid, linolenic acid, oleic acid, stearic acid, 12-hydroxyoctadec-cis-9-enoic acid (ricinoleic acid) and palmitic acid	Bael seed oil	Nutrient recycling	[46]
Essential oils	3-Isothujanol, α-humulene, α-phellandrene, δ-carene, 4-terpineol, linalool, α-terpinyl isobutyrate, trans-2-hydroxy cinnamic acid, γ-curcumene, α-muurolene, β-bisabolene, α-cubebene, γ-elemene, isosylvestrone, α-pinene, β-ocimene, γ-terpinene, terpenolene, α-terpineol, γ-cadinene, thuj-3-en-10-al, δ-elemene, γ-muurolene, β-myrcene, valencene, β-selinene and β-bisabolol	Leaves, stem and roots	Antifungal, antioxidant, antibacterial and hepatoprotective	[22, 37]
Miscellaneous	Allo-imperatorin, anthocyanins, auraptine, β-sitosterol, carotene, dimethoxy coumarin, γ-sitosterol, hamycin, imperatorin, lembamide, lupeol, luvangetin, marmin, marmesin, oxalic acid, psoralin, scopoletin, skimmlamine, skimmianine, umbelliferone, xanthotoxin and α-amyrin	Whole plant	Anticancer, anti-methamphetamine, sedative, hypnotic, analgesic, anticonvulsive, antipyretic, hypothermic, antidiuretic and antimalarial	[50, 51]

**Table 6:** Important bioactive substances in AM including their actions and known impacts.

Bioactive compound	Category	Mechanism of action	Reported effects	Reference
Marmelosin	Coumarin	Antioxidant; AChE inhibition; anti-inflammatory	Neuroprotective; cognitive improvement in AD	[52]
Aegeline	Alkaloid	SNARE protein mimicry; α-synuclein toxicity suppression	Parkinson's disease prevention	[7]
Imperatorin	Coumarin	PPAR-γ activation; NO inhibition	Anticonvulsant activity in epilepsy	[8, 53]
Chlorogenic acid	Phenolic acid	Antioxidant activity; ROS scavenging	Neuroprotection; reduced oxidative stress	[54, 55]
Flavonoids (e.g., rutin)	Flavonoid	ROS scavenging; lipid peroxidation inhibition	Neuroprotection; reduced depression-like behaviour	[56]
Skimmianine	Alkaloid	Not specified	Antioxidant, anti-inflammatory	[25]
Linalool	Terpenoid	Not specified	Anti-inflammatory and ROS scavenging, contributing to neuroprotection and cognitive enhancement	[57]
Antidiarrheal activity	—	Antibacterial; calcium channel blocking compounds	Significant reduction in faecal output; inhibition of bacterial colonization (*E. coli*, *S. dysenteriae*)	[33]
Antioxidant activity	—	Free radical scavenging; ROS inhibition	Significant scavenging of DPPH, NO, H_2_O_2_; antioxidant potential	
Antidiabetic activity	—	Insulin regulation; glucose metabolism improvement	Reduced blood glucose; increased insulin levels; improved body weight	
Hepatoprotective activity	—	Antioxidant; restoration of liver enzymes	Reduction in liver enzyme levels (ALT, AST and ALP) in hepatotoxicity models	
Radioprotective activity	—	Protection against radiation-induced damage; antioxidant	Increased survival rates postradiation exposure	
Anticancer activity	—	Antioxidant; antiproliferative; chemopreventive	Tumour reduction in animal models; antiproliferative effects on breast cancer cells	

**Table 7:** Chemical compounds responsible for neuroprotective activity with chemical structure and mechanism of action.

Neurological/behavioural disorders	Chemical compound	Mechanism of action	Structure	Reference
Alzheimer's disease	α-Tocopherol, β-carotene, glutathione, ascorbic acid, total flavonoids, total polyphenols	AChE inhibitory properties and antioxidative activities.	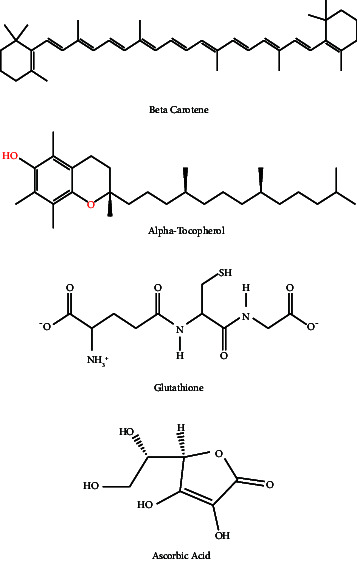	[55, 61]

Parkinson's disease	Aegeline	Aegeline, a ySec22p mimic, has potential in inhibiting yeast apoptosis in response to α-synuclein aggregation and Bax expression. The cellular mechanisms linked to neurodegeneration are the focus of this mechanism's investigation as a possible PD therapy. Validating its safety and efficacy will require more study and clinical studies.	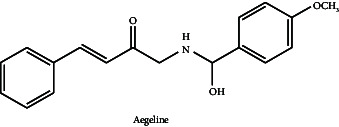	[7]

Epilepsy	Imperatorin (coumarins derivative)	This protective effect could be due to activation of PPAR-c activation, which suppresses NO generation further.	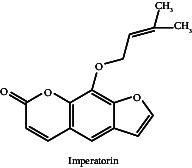	[8]

Anxiety	AM leaves extract	Not specified		[137]

Depression	• Imperatorin • Aegeline	• Male prenatally stressed offspring showed higher 5-HT concentration and antidepressive-like effects, indicating that imperatorin may be therapeutically useful in preventing depressive-like behaviour in adolescence. • Aegeline could have a protective impact by reducing IL-6 levels, oxidative and nitrosative stress, and MAO-A hyperactivity.	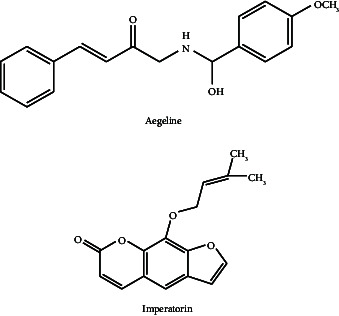	[9, 143, 144]

## Data Availability

Data are contained within the article.
